# Chrysin Induces Antidiabetic, Antidyslipidemic and Anti-Inflammatory Effects in Athymic Nude Diabetic Mice

**DOI:** 10.3390/molecules23010067

**Published:** 2017-12-28

**Authors:** Juan José Ramírez-Espinosa, Johann Saldaña-Ríos, Sara García-Jiménez, Rafael Villalobos-Molina, Gabriela Ávila-Villarreal, Angélica Nallelhy Rodríguez-Ocampo, Germán Bernal-Fernández, Samuel Estrada-Soto

**Affiliations:** 1 Instituto de Ciencias Biomédicas, Universidad Autónoma de Ciudad Juárez, Juárez 32310, Chihuahua, Mexico; jjre9785@gmail.com; 2 Facultad de Farmacia, Universidad Autónoma del Estado de Morelos, Cuernavaca 62209, Morelos, Mexico; srje_ff@uaem.mx (J.S.-R.); saragarcia@uaem.mx (S.G.-J.); 3 UBIMED, FES-Iztacala, Universidad Nacional Autónoma de México, Tlalnepantla 54090, Estado de México, Mexico; villalobos@campus.iztacala.unam.mx; 4 Unidad Académica de Ciencias Químico Biológicas y Farmacéuticas, Universidad Autónoma de Nayarit, Tepic 63000, Nayarit, Mexico; gaby.avila@uan.edu.mx (G.Á.-V.); angelica.rodriguez@uan.edu.mx (A.N.R.-O.); 5 Centro Nayarita de Innovación y Transferencia de Tecnología “Unidad especializada en I+D+*i* en Calidad de Alimentos y Productos Naturales”, Universidad Autónoma de Nayarit, Tepic 63173, Nayarit, Mexico

**Keywords:** antidyslipidemic, chrysin, diabetes, inflammation, nude mice

## Abstract

Extensive knowledge of diabetes and its complications is helpful to find new drugs for proper treatment to stop degenerative changes derived from this disease. In this context, chrysin (5,7-dihydroxyflavone) is a natural product that occurs in a variety of flowers and fruits with anti-inflammatory and antidiabetic effects, among others. Thus, a diabetic model in athymic nude mice was developed and used to establish the ability of chrysin to decrease the secretion of pro-inflammatory cytokines. Also, it was determined the acute (50 mg/kg) and sub-acute (50 mg/kg/day/10 days) antidiabetic and antihyperlipidemic activities after the period of time treatment. Results indicate that chrysin has significant acute antihyperglycemic and antidiabetic effects in nude diabetic mice (*p* < 0.05). Moreover, triglyceride blood levels were reduced and IL-1β and TNF-α were diminished after 10 days’ treatment compared with control group (*p* < 0.05). In conclusion, it was found that chrysin could produce similar effects as metformin, a drug used for the treatment of diabetes, since both test samples decreased glucose and triglycerides levels, they impaired the generation of pro-inflammatory cytokines involved in the development of diabetes and its consequences, such as atherosclerosis and other cardiovascular diseases.

## 1. Introduction

Type 2 diabetes is a chronic disease characterized by insulin resistance and deficiency of insulin secretion, which could be present at any age and affects more than 300 million people worldwide [1]. Despite its epidemiology, an efficient and safety drug for diabetes treatment is still to be developed due to multiple adverse reactions and drug interactions of current treatments [2]. Furthermore, the development of new drugs that could control and reduce diabetes complications by deploying other effects, such as pro-inflammatory cytokines, could be useful to improve the outcome of the disease [3]. In this context, natural products are an important source to find a desirable drug for diabetes and the regulation of inflammation, for instance chrysin (5,7-dihydroxyflavone) is a natural occurring flavonoid present in the aerial parts of *Oroxylum*, *Chamomille* and *Passiflora* genus and also it is abundant in honey and propolis. Chrysin possesses antidiabetic, anti-inflammatory and antioxidant properties and is widely used as nutritional supplement for body-building for its aromatase inhibition effect [4]. Chrysin also has been described as an antidiabetic compound with cardiac and hepatic protective effects, due to its increase of antioxidant enzymes and antidyslipidemic activities in experimental diabetic rats [5]. This antidiabetic action could be related to the inhibition of 11β-hydroxysteroid dehydrogenase type 1, which lowered cortisol production and enhancing insulin sensitivity [6]. Moreover, some studies have established the anti-inflammatory capabilities of this compound, especially by inhibiting the production of pro-inflammatory molecules from COX-2 activity, NF-κB, TNF-α and IFN-γ [7]. Chrysin has an important role on interleukin production, i.e. it has been established that, in pro-inflammatory conditions derived from LPS stimulation or ischemic/reperfusion events, the levels of IL-1α, IL-1β, IL-6, IL-8 and IL-12 are diminished [8]. These data prompted us to use athymic mice as a model to study the antidiabetic and anti-inflammatory activities of chrysin [9].

## 2. Results and Discussion

During current investigation, the effects of chrysin on glucose metabolism and inflammation in athymic nude mice were evaluated. The action of this flavonoid was evaluated in normoglycemic athymic nude mice to determine its antihyperglycemic effect in glucose and sucrose tolerance tests.

### 2.1. Antihyperglycaemic Activity

**Figure 1:**
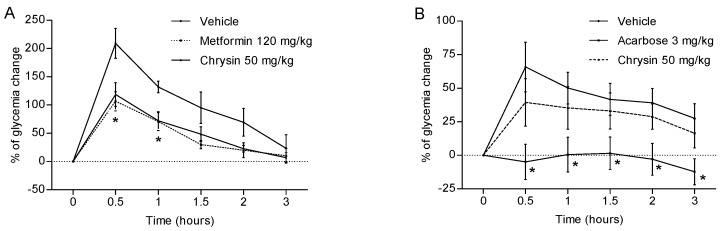
Effect of chrysin (50 mg/kg) on blood glucose levels after single oral administration of 2 g/kg of (**A**) glucose and (**B**) sucrose in athymic nude normoglycaemic mice. Each plot represents the means ± SEM, *n* = 8; * *p* < 0.05.

The acute evaluation showed that chrysin (50 mg/kg) has an antihyperglycaemic effect, similar to that observed for metformin at 120 mg/kg, diminishing to one half of blood glucose levels during the experiment (Figure 1A). The difference was significant (*p* < 0.05) at 0.5 and 1 h, in comparison with the control group. The fact that chrysin showed significant reduction of hyperglycaemic peak at 0.5 h suggests that this bioactive compound is acting, in part, by inhibition of glucose transporters SGLT-1 or GLUT2 [10]. Also, antihyperglycaemic action showed by chrysin was observed at 0.5 h and lasted all the experiment (as metformin does) and it might be related with a better organism’s glucose metabolism and glucose uptake after intestinal glucose absorption. This is a probable consequence of insulin sensitizing due to chrysin induced PPAR-γ activation [11] and 11β-HSD1 inhibition [6]. On the other hand, chrysin does not have an important effect on glucose absorption or metabolism after sucrose administration, which was expected since previous data shown that chrysin has no glucosidase inhibitory activity, neither induce changes on sucrose tolerance test curves (Figure 1B) [12]. The control group treated with acarbose had lower values (*p* < 0.05) than the vehicle group, across the experiment (Figure 1B).

### 2.2. Glucose Determination and Biochemical Analysis after Sub-Acute Treatment with Chrysin

**Figure 2:**
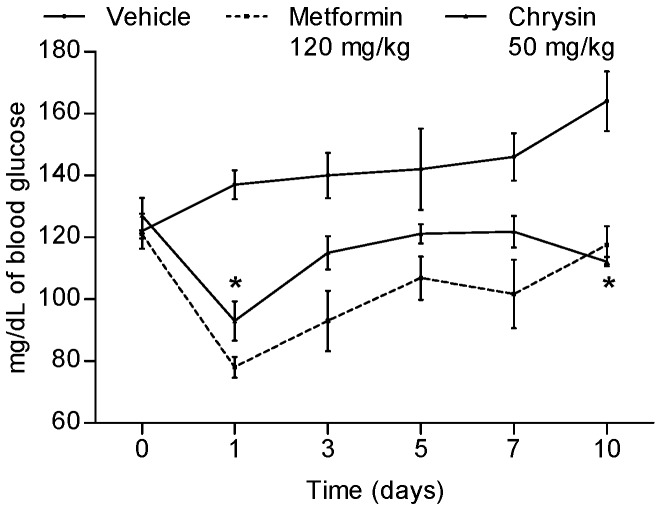
Antidiabetic effect of subacute intragastric administration of chrysin (50 mg/kg), metformin (120 mg/kg) and vehicle in streptozotocin–nicotinamide diabetic nude mice. Each plot represents the means ± SEM, *n* = 8; * *p* < 0.05.

Previous works revealed that chrysin has significant acute antidiabetic action in non-insulin dependent diabetic murine models [5,6,13] and it was also demonstrated its antihyperglycemic action. Thus, we determined that the sub-acute antidiabetic, antihyperlipidemic and anti-inflammatory activities in a new diabetic mice model were induced by nicotinamide/streptozotocin intraperitoneal administration, allowing a hyperglycemic state derived from pancreatic dysfunction by β-cells damage in nude mice. Once fasting glucose was higher than 120 mg/dL and symptoms as weight loss and polyuria were observed, we proceeded to a sub-acute assay. Over 10 days three different diabetic athymic nude mice groups were administered with either 50 mg/kg of chrysin, vehicle (control) or metformin (120 mg/kg, positive control); blood drops from tail tip were used for glucose monitoring. A diminution of glycaemia in the chrysin group was observed in comparison with the control group (Figure 2) being significant at days 1 and 10 (*p* ≤ 0.01); however, blood glucose level was controlled along all treatment times compared with the control group, whose glycaemia increased gradually until the end of the experiment. Results from glucose tolerance test correlate with previous studies in normal and diabetic Wistar rats, indicating that chrysin acts as an insulin sensitizer [6,11]. It is important to point out that mice treated with metformin showed a tendency to lose weight, similar to vehicle, while chrysin treatment halted such trend, which could be related to antioxidant or anti-inflammatory activities.

For a better understanding of chrysin effects on energy metabolism, blood glucose and lipid profile were determined at 11 days after treatment. Results of this analysis showed that, when compared with the control group, chrysin-treated mice diminished glycaemia (*p* < 0.05) to lower level than metformin-treated mice, suggesting that chrysin could be useful as antidiabetic agent for successful treatment of diabetes over a long period of time as insulin sensitizer [13]. On the other hand, in metformin and chrysin groups, lipid profile displayed important changes, LDL was augmented and VLDL and triglycerides (TG) were diminished (Table 1) (*p* < 0.05). These observations suggest that fatty acids metabolism is being modified towards LDL synthesis due to glycolysis metabolites diminution, maybe by PPAR-γ activation [11] and 11β-HSD1 inhibition [6] (Table 1). Total cholesterol and HDL levels were not significantly modified by chrysin treatment.

### 2.3. Anti-Proinflammatory Effect

After sub-acute treatment with test samples, serum IL-1β, IL-6 and TNF-α profile was measured. Thus, it is important to indicate that athymic nude mice do not have an adaptive immune response through T cells to external stimuli or inflammatory elements [14]; consequently, the changes observed in the cytokines profile during experimentation must be due to the diabetic state, which induced a pro-inflammatory condition, or probably by treatment effects on macrophages.

On the other hand, the effects of hyperglycaemia on oxidative stress on macrophages and pro-inflammatory cytokines has been studied in streptozotocin-induced diabetic rats and mice; such studies suggest that hyperglycaemia induces the macrophage activation by transcription factor FoxO1 expression, which activates NF-κB and thus increased production and release of IL-1β and TNF-α [15], through TLR-4 that recognize selective host lipids (saturated fatty acids), which playing an important role in the pathogenesis of not infectious, inflammatory diseases of lipid dysregulation such as insulin resistance and obesity. In fact, mice lacking TLR-4 are protected against insulin resistance [16,17]. It should be noted that hyperglycaemia induces liver and adipose tissue macrophage infiltration, generating increased local concentrations of IL-1β, TNF-α, iNOS and COX-2 for JNK/NF-κB pathway activation [18]. It also has been proposed that hyperglycaemia promotes IL-1β released from pancreatic β cells and induce their death trough apoptosis by activating NF-κB/FAS pathway [19]. Hence, this might indicate how the levels of IL-1β and TNF-α are increased in the absence of adipogenic processes, or in the presence of T cells in diabetic athymic nude mice.

**Figure 3:**
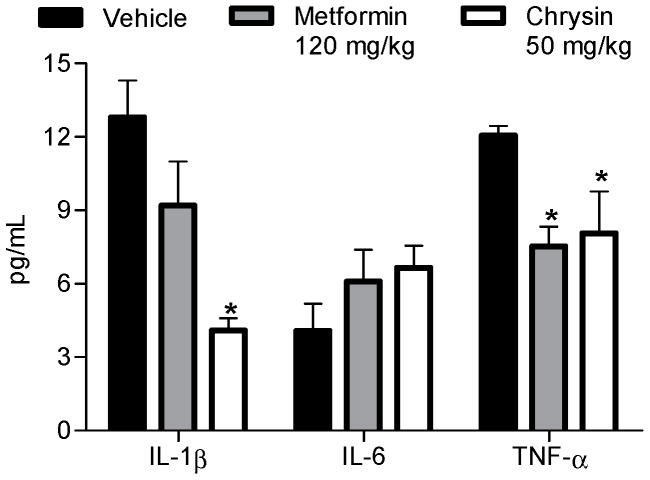
Effect of chrysin (50 mg/kg) on blood pro-inflammatory cytokines profile after ten days of treatment in streptozotocin–nicotinamide diabetic nude mice. Each plot represents the means ± SEM, *n* = 8; * *p* < 0.05.

Now, the role of macrophages infiltrated in the liver, as the main contributor of observed IL-1β and TNF-α levels, was corroborated by the fact that metformin significantly decreased both cytokines (Figure 3); since, in the liver, metformin decreases expression of FoxO1 and stops both via PEPCK/G6P as JNK/NF-κB through the AMPK/SHP route [20]. In this context, metformin and chrysin were capable to reduce the pro-inflammatory cytokines IL-1β and TNF-α serum concentration and production, probably by the effects of both compounds in the cellular pathway that improves the metabolism of carbohydrates (Figure 3) and/or by the mechanism above described [18,20]. Finally, IL-6 level was not diminished, probably because the pro-inflammatory stimulus of T cells or obesity development are absent. However, previous work described that, in models of vascular disease or obesity, metformin or chrysin decreased the levels of IL-6 [21].

Finally, our findings are relevant and with additional clinical studies could be useful for the human health. Also, it is necessary to develop more preclinical research investigation to know about its toxicological and pharmacokinetic features. On the other hand, studies about chrysin indicate that it is found in considerable amounts in murine blood plasm [22] which can justify its pharmacological effects. Also, its log P = 2.94 and Polar surface area (PSA) = 70.66 [23] suggest that Chrysin crosses the cell membrane by passive diffusion, which guarantees its presence in the blood stream. Finally, it is complicated to establish how much of chrysin is present in the murine blood and in consequence, we cannot translate to human, however, we are planning to develop the pharmacokinetic studies in a murine model and then do it in humans to get more information about it. Dose studied in current work showed significant effects in nude mice, however, it is necessary to do dose-response studies to establish the DE_50_ and LD_50_ for determine the therapeutic window and extrapolate to humans for clinical studies.

## 3. Materials and Methods

### 3.1. Chemical and Drugs

Chrysin (purity 97%), streptozotocin (purity ≥ 75% of α-anomer), tween 80 (cell culture grade), acarbose (purity ≥ 95%) and metformin (analytical grade) were purchased from Sigma-Aldrich Co. (Saint Louis, MO, USA). Also, glucose (reactive grade), sucrose (reactive grade) and nicotinamide (reactive grade) were acquired from Merck Co. (Kenilworth, NJ, USA).

### 3.2. Animals

Male athymic nude mice were used. All animals with initial body weight of 25–30 g, and 8 weeks old were housed under sterile laboratory conditions with 12 h day-night cycle, 25 ± 2 °C and 45–65% of humidity. All animal procedures were conducted according to Mexican Federal Regulations for Animal Experimentation and Care (NOM-062-ZOO-1999, SAGARPA, México City, Mexico) and approved by the institutional Animal Care and Use Committee (UNAM, February 2015) and based on US National Institute of Health publication #85-23 (1985). Before the acute procedures all animals were fasted during 16 h with water *ad libitum*.

### 3.3. Oral Glucose and Sucrose Tolerance Tests

Three groups of mice (*n* = 8) were formed for per os administration of 0.1 mL of vehicle solution (Tween 80 at 10%), positive control (metformin 120 mg/kg or acarbose 3 mg/kg, dissolved in saline solution) or chrysin (50 mg/kg, dissolved in vehicle). Thirty minutes after test samples administration, a dose of 2 g/kg of substrate (glucose or sucrose dissolved in drinking water) was administered orally to each mouse. Blood samples were collected from the tail tip at 0 (before sample administration), 0.5, 1, 1.5, 2 and 3 h. after substrate delivery.

### 3.4. Induction of Hyperglycaemic Condition

For diabetes induction, 24 athymic nude mice were administered intraperitoneally (ip) with nicotinamide (65 mg/kg) dissolved in saline solution and 30 min later were injected ip with streptozotocin (110 mg/kg) dissolved in citrate buffer pH 4.5. After 15 days those mice with fasting glucose above 120 mg/dL were considered hyperglycaemic.

### 3.5. Orally Sub-Acute Treatment and Glucose Determination

Three groups of hyperglycaemic athymic nude mice (*n* = 8) were orally treated with 0.1 mL of vehicle solution (Tween 80 at 10%), metformin (120 mg/kg) or chrysin (50 mg/kg) for ten days. Blood glucose concentration was monitored by tail drops using an Accu-Check Performa glucometer (Roche Diagnostics^®^, Indianapolis, IN, USA) 3 and 24 h, respectively, after administration at days 1, 3, 5, 7 and 10 to establish hypoglycemic effect.

### 3.6. Determination of Blood Parameters

Blood from sub-acute treated mice were obtained by cardiac puncture and centrifuged for 15 min at 3500× *g*, the serum was obtained and analyzed by enzymatic-photocolorimetric methods for glucose and lipids quantification using Roche Diagnostics reagents for a Cobas c-111 (Roche, Basel, Switzerland) apparatus [24].

### 3.7. Quantification of IL-1β, IL-6 and TNF-α Cytokines

The serum obtained from blood samples was used for cytokines quantification assay. A MILLIPLEX MAP kit (Millipore, Burlington, MA, USA) was used to measure the concentrations of IL-1β, IL-6 and TNF-α, according to the manufacturer’s instructions. Plate was read using the Luminex 200 system and analytes’ concentrations were calculated using xPONENT software (Luminex, Austin, TX, USA) [25].

### 3.8. Statistical Analysis

Data are presented as the arithmetic mean ± standard error of mean (SEM). Differences between groups were analyzed by one-way ANOVA calculations with Bonferroni post-hoc analysis using the GraphPad Prism 5.01 software (Graph Pad Software, San Diego, CA, USA). A *p*-value < 0.05 was considered statistically significant.

## 4. Conclusions

This work contributes to the understanding of the relationship between hyperglycemia with pro-inflammatory cytokines and the response to different treatments (metformin and chrysin) in a model of diabetic athymic nude mice. Furthermore, it was found that chrysin could produce similar effects as metformin, a drug used for the treatment of diabetes, since both test samples decreased glucose levels and they impaired the generation of pro-inflammatory cytokines involved in the development of diabetes and its consequences, such as atherosclerosis and other cardiovascular diseases.

## Figures and Tables

**Table 1:** Changes in blood parameters after ten days of treatment with chrysin. (*) variance of mean significance ≤ 0.05. GLU: glucose, CHO: cholesterol, TG: triglycerides, HDL: high density lipoprotein, LDL: low density lipoprotein, VLDL: very low density lipoprotein.

Parameter (mg/dL)	Vehicle (1 mL)	Metformin (120 mg/kg)	Chrysin (50 mg/kg)
GLU	172 ± 2	153 ± 13	132 ± 10 *
CHO	210 ± 4	208 ± 7	201 ± 7
TG	223 ± 13	167 ± 12 *	167 ± 14 *
HDL	101 ± 3	118 ± 9 *	104 ± 4
LDL	27 ± 1	43 ± 4 *	42 ± 5 *
